# Association of F-53B Nephrotoxicity with Oxidative Stress-Mediated Mitochondrial Dysfunction and Altered Autophagy–Apoptosis Crosstalk

**DOI:** 10.3390/biom16070938

**Published:** 2026-06-24

**Authors:** Bitong Li, Dongling Liu, Zhiying Qiu, Yaojian Zheng, Yue Wu, Lina Zhang, Ran Li, Cuiqing Liu, Qinghua Sun, Xiang Zeng

**Affiliations:** 1 School of Public Health, Zhejiang Chinese Medical University, 548 Binwen Road, Hangzhou 310053, China; 2 School of Basic Medical Science, Zhejiang Chinese Medical University, 548 Binwen Road, Hangzhou 310053, China; 3 Zhejiang International Science and Technology Cooperation Base of Air Pollution and Health, 548 Binwen Road, Hangzhou 310053, China

**Keywords:** F-53B, nephrotoxicity, oxidative stress, mitochondrial dysfunction, autophagy, apoptosis

## Abstract

6:2 chlorinated polyfluorinated ether sulfonate (F-53B, also known as 6:2 Cl-PFESA) is a major alternative to perfluorooctane sulfonate (PFOS) and a widespread environmental pollutant with potential public health hazards. However, its nephrotoxic effects and underlying molecular mechanisms remain poorly understood. This study investigated renal injury induced by environmentally relevant concentrations of F-53B and delineated the mechanistic cascade using a mouse model combined with quantitative proteomic and molecular biological approaches. Male C57BL/6 mice were exposed to 0, 4, 40, and 400 μg/L F-53B for 4 weeks. F-53B exposure led to significant renal dysfunction, histopathological damage, elevated renal injury biomarkers, and pronounced oxidative stress in a dose-dependent manner. A proteomic comparison of the 0 μg/L versus 400 μg/L groups identified 276 differentially expressed proteins that were strongly enriched in oxidative phosphorylation, autophagy, and apoptosis pathways, with cytochrome c oxidase subunit 7b (Cox7b) serving as a core downregulated hub molecule. Further validation confirmed that F-53B triggered overt mitochondrial structural damage, impaired respiratory chain complex assembly, aberrant ATP production, and disturbed mitochondrial dynamics. Consequently, excessive autophagy activation and mitochondrial-mediated apoptosis were simultaneously stimulated in renal tissues. Notably, although statistically significant, the alterations induced by F-53B were generally mild in magnitude. Collectively, our findings demonstrate that F-53B induces nephrotoxicity through a sequential pathological cascade. This study provides novel mechanistic insights into F-53B-elicited renal injury and highlights the potential health risks of this emerging per- and polyfluoroalkyl substance (PFAS) alternative.

## 1. Introduction

Per- and polyfluoroalkyl substances (PFAS) are synthetic fluorinated compounds widely used in industrial and consumer products since the 1940s. Owing to their environmental persistence, bioaccumulation potential, and multiple organ toxicity, PFAS have attracted global regulatory and scientific concern [1]. As two typical legacy PFAS congeners, perfluorooctanoic acid (PFOA) and perfluorooctane sulfonate (PFOS) have been the most extensively studied legacy PFAS and have been strictly restricted or banned in many countries [2,3]. In accordance with national environmental management requirements, China officially banned the production and commercial application of PFOS in 2019, with only a small number of special industrial scenarios granted exemption approval by the Ministry of Ecology and Environment [4]. As a primary alternative to PFOS products, 6:2 chlorinated polyfluorinated ether sulfonate (F-53B, 6:2 Cl-PFESA) has been widely used as a mist suppressant in the electroplating industry [5]. Consequently, F-53B has become ubiquitous in environmental matrices, including aquatic systems (0.56–78.5 ng/L) [6], urban wastewater (0.02–209 ng/g) [7], and human serum (1.55–8.64 ng/mL) [8]. Notably, extremely high F-53B concentrations ranging from 65 to 112 μg/L have been detected in raw electroplating wastewater, and the concentration in partial downstream aquatic environments even exceeds 7 μg/L, posing potential persistent threats to aquatic organisms and human health via drinking water and dietary exposure pathways [5].

Recent evidence suggests that F-53B exerts hepatotoxicity, intestinal toxicity, neurotoxicity, and reproductive toxicity. However, its nephrotoxicity and detailed molecular mechanisms remain largely unexplored. The kidney is a vital excretory organ responsible for systemic water–electrolyte balance, metabolic waste elimination, and endocrine function. Due to its high perfusion rate, rich mitochondrial content, and active metabolic activity, the kidney is particularly vulnerable to environmental toxicants, including PFAS. Accumulating evidence links PFAS exposure to decreased glomerular filtration function, irreversible renal tubular damage, increased incidence of chronic kidney disease and even renal cancer [9]. For instance, occupational workers with high PFOA exposure exhibited elevated levels of renal impairment biomarkers, including creatinine (CREA) and uric acid (UA) [10]. A cross-sectional cohort study targeting adolescents illustrated that serum PFAS concentrations among Korean children were twice as high as those in American peers, and circulating PFAS levels were negatively correlated with estimated glomerular filtration rate (eGFR) [11]. In vivo animal experiments further validated renal histological damage induced by short-chain and long-chain PFAS: chronic oral administration of perfluorohexanoic acid (PFHxA) induced renal papillary necrosis and tubular degeneration in female rats [12], while perfluoroundecanoic acid (PFUnDA) exposure triggered interstitial inflammatory infiltration, renal hemorrhage and tubular dilatation in mice [13]. Nevertheless, the specific mechanisms underlying PFAS-induced nephrotoxicity remain to be fully clarified.

Excessive reactive oxygen species (ROS) accumulation induced by exogenous toxicants can destroy mitochondrial structure and impair mitochondrial respiratory function in renal tubular epithelial cells [14]. Damaged mitochondria further promote massive ROS release, thereby forming a positive feedback vicious cycle to aggravate renal cellular oxidative damage. Mitochondrial dysfunction also serves as a central hub regulating autophagy and apoptosis, two critical processes governing cell fate. Under stress conditions, moderate autophagy acts as an adaptive survival mechanism by removing damaged organelles, while excessive or dysregulated autophagy may trigger programmed cell death. Pretreatment with autophagy inhibitors in rats alleviates the severity of renal ischemic reperfusion injury [15]. Apoptosis, a form of programmed cell death, is directly activated by mitochondrial injury and plays a key physiological role in repairing damaged cells and maintaining the homeostasis of cells [16], and crosstalk between autophagy and apoptosis plays a critical role in the progression of renal diseases. Due to its structural similarities with PFOS, the F-53B raises concerns for nephrotoxicity, and emerging evidence indicates that exposure to F-53B may induce renal fibrosis, possibly via mechanisms including oxidative stress, inflammation and activation of the TGF-β1/Smad3 signaling pathways [17]. However, the exact molecular mechanisms remain unclear and require further investigation to fully understand the long-term impact of F-53B on renal health.

## 2. Materials and Methods

### 2.1. Chemicals and Reagents

F-53B was purchased from Shanghai Maikun Chemicals Co., Ltd., Shanghai, China. (CAS# 73606-19-6, purity ≥ 99%). Accurately weigh 10 mg of F-53B powder and dissolve the powder in 1 mL dimethyl sulfoxide (DMSO) to prepare a 10 mg/mL stock solution. This stock was further diluted with double-distilled water to yield working concentrations for subsequent experiments. To avoid solvent toxicity, the final DMSO concentration in all treatment groups was controlled at <0.001% (*v*/*v*).

### 2.2. Animals in Experimental Design

C57BL/6 mice are the most widely used in toxicology, environmental toxicology and renal toxicity research. This strain has a clear genetic background, stable physiological characteristics, and mature feeding and modeling protocols, rendering the extrapolation of experimental findings more clinically valuable. Six-week-old male specific pathogen-free (SPF) C57BL/6 mice were purchased from Shanghai BK Company Co., Ltd., Shanghai, China. Prior to the formal experiment, mice were acclimatized for one week in a climate-controlled room maintained at 23 °C ± 2 °C temperature, 55% ± 5% humidity, and a 12 h light and dark cycle, with free access to standard rodent food and water. Subsequently, mice were randomly allocated to four treatment groups: the control group received deionized water only; the low group was administered deionized water containing 4 μg/L F-53B; the medium group received deionized water containing 40 μg/L F-53B; and the high-dose group received deionized water containing 400 μg/L F-53B. The choice of the dose was based on previously reported internal exposure levels of the general population and occupational workers accompanied by F–53B concentration in fresh water [18]. Body weight and water consumption were monitored and recorded. Fresh drinking water and food were replenished every twenty-four hours. After four weeks of exposure, mice were humanely euthanized, and samples of serum and renal tissue were collected for further analyses. This work has received approval for research ethics from Zhejiang Chinese Medical University (Approval No: IACUC-20240624-10; approved date: 22 August 2024), and a proof/certificate of approval is available upon request.

### 2.3. Histological Analysis

Renal tissue samples were fixed in 4% paraformaldehyde solution for 48 h. Subsequently, the specimens underwent gradient ethanol dehydration, paraffin embedding, and continuous sectioning at a thickness of 4 μm. Routine haematoxylin and eosin (H&E) staining was performed on tissue sections for general morphological observation of renal lesions. Periodic acid-Schiff (PAS) staining was additionally conducted to assess the structural integrity of brush borders in renal proximal tubules. All stained tissue sections were observed and imaged under an optical microscope. Histopathological scores were performed independently by two blinded investigators from the treatment groups. For H&E-stained sections, renal tubular injury was evaluated using a 0–3 semi-quantitative scoring system based on the area of damaged renal tubules: score 0, normal renal tissue with no obvious morphological damage; score 1, tubular damage area ≤25%; score 2, tubular damage area ranging from 26% to 50%; score 3, tubular damage area ranging from 51% to 75% [19]. In PAS, the loss of the brush border in the proximal tubule glycocalyx was scored from 0 to 4. The grading criteria were defined as follows. score 0: no damage or intact structure; score 1: 1–10% loss; score 2: 11–25% loss; score 3: 26–75% loss; score 4: >75% loss [20].

### 2.4. Ultrastructure Observation of Tissue by Transmission Electron Microscopy (TEM)

Immediately after removal from the body, small pieces of cortical tissue were preliminarily cut into a culture medium supplemented with 2.5% glutaraldehyde solution. The tissue blocks with a volume of approximately 1 mm^3^ were then fixed in fresh 2.5% glutaraldehyde for primary fixation lasting for 2 to 4 h, followed by three rinses in phosphate-buffered solution (PBS). Subsequently, the samples were post-fixed in 1% osmic acid and sealed in the dark for 1 to 2 h, and rinsed three times again with PBS. Dehydration was carried out using a graded series of ethanol solutions, followed by pure acetone. The dehydrated samples were then infiltrated with a 1:1 mixture of acetone and embedding resin at room temperature for 1 h. Afterwards, a mixed solution containing embedding resin and acetone (volume ratio = 3:1) was used for secondary infiltration for 3 h. Finally, the tissue samples were immersed in pure embedding resin and placed overnight at room temperature under dark conditions.

The following day, the tissues were transferred to a fresh, clean resinous substrate and placed in a press-fed furnace set at 70 °C for 24 h. The resulting blocks of resin were stored for future use. Subsequently, ultrathin sections were prepared and double-stained with uranyl acetate and lead citrate and finally examined by TEM to obtain an ultrastructural image of the kidney tissue [21].

### 2.5. Serum Biochemical Analysis

The blood samples were allowed to clot for one hour at room temperature and then centrifuged at a rate of 3000 rpm for 15 min at a temperature of 4 °C to separate the serum. The supernatant (serum) was collected and stored at −80 °C until analysis [21]. The serum levels of biomarkers of renal function, such as creatinine (CREA), blood urea nitrogen (BUN) and uric acid (UA), were measured by an automatic biochemical analyzer (Hitachi, Ltd., Tokyo, Japan).

### 2.6. Measurement of Oxidative Stress Markers

The SOD and MDA levels were determined using commercially available kits (Nanjing Jiancheng Institute of Bioengineering, Nanjing, China). SOD activity measurement relies on the inherent capacity of SOD to scavenge superoxide anion radicals. In the assay kit, free radicals are first generated and subsequently react with chromogenic substrates such as WST-1 or NBT. Higher SOD activity eliminates more free radicals, resulting in fainter color development. MDA quantification is based on the reaction between MDA and thiobarbituric acid (TBA). Under heated acidic conditions, MDA conjugates with TBA to form a red adduct; greater color intensity corresponds to higher MDA concentrations. Both assays are colorimetric methods. Fresh tissue of 20 mg was added to physiological saline at a 1:9 ratio, homogenized and centrifuged at 4000 rpm. The whole process strictly followed the instructions of the kit [19].

### 2.7. Detection of ATP Content

ATP levels in renal tissues were measured with the Shanghai Beyotime Biology ATP assay kit (Shanghai Beyotime Biotechnology Co., Ltd., Shanghai, China). This assay kit is developed based on the biochemical principle that firefly luciferase requires ATP as an energy source to catalyze luciferin into luminescent products. With excess dosages of both firefly luciferase and luciferin, luminescence intensity is linearly proportional to ATP concentration within a certain concentration range. This detection method is categorized as a bioluminescence assay. Briefly, the fresh tissues were homogenized in ATP lysate buffer (300 μL) under ice-bath conditions. The homogenates were centrifuged at 4 °C, and the supernatant was collected for immediate analysis according to the kit protocol [19].

### 2.8. Quantitative PCR (QPCR) Analysis

The total RNA was extracted from the kidney tissues with the aid of the TRIZOL reagent. RNA has been reverse-transcribed to complementary DNA (cDNA) by means of the Takara reverse-transcription kit (Takara Bio, Kusatsu, Japan). The qPCR was carried out using the SYBR Green master mix in a QuantStudio 7 Flex instrument (Applied Biosystems, Foster, CA, USA) [22], for real-time detection of gene expression levels. The thermal cycling conditions were as follows: initial denaturation at 95 °C for 10 min, followed by 40 cycles of 95 °C for 15 s and 60 °C for 1 min. The relative mRNA expression levels of target genes were normalized to β-actin and calculated using the 2^−ΔΔCt^ method. The primer sequences of target genes used are listed in Table 1.

### 2.9. Western Blot

Total protein was extracted from renal tissues by RIPA lysis buffer, and the bicinchoninic acid (BCA) concentration was determined by the BCA test. The proteins were separated by SDS-PAGE polyacrylamide gel electrophoresis (SDS-PAGE) and transferred to a polyvinylidene fluoride (PVDF) membrane via wet transfer. The membrane was blocked with 5% skimmed milk and incubated overnight with corresponding primary antibodies at 4 °C. Following three washes (10 min each) with TBST buffer (1×), the membrane was incubated with a horseradish peroxidase (HRP)-conjugated secondary antibody for 1 h at room temperature. After additional washes, protein bands were visualized using an enhanced chemiluminescence (ECL) substrate (Bio-Rad Laboratories, Inc., Hercules, CA, USA) and imaged with a chemiluminescence detection system [22]. Detailed antibody information is provided in Table 2.

### 2.10. Immunohistochemical Staining

The paraffin sections were dewaxed and rehydrated, followed by antigen extraction. The endogenous peroxidase activity was blocked, and serum was used to block the activity. After adding the prepared primary antibody to the sections, the blocking solution was gently removed. The sections were then placed on a flat surface in a wet chamber and incubated overnight at 4 °C. The following day, secondary antibody was administered, followed by DAB staining. After a slight drying of the sections, fresh chromogenic DAB solution was applied in the indicated circles. The time to stain was carefully monitored under the microscope, and positive staining was brownish yellow. The staining process was completed by rinsing the sections with tap water. The nuclei were then stained with hematoxylin before dehydration and assembly [22]. Quantitative analysis of the positive areas was performed by immunohistochemical staining techniques using the Saiviewer software 2.4.0.

### 2.11. Terminal Deoxynucleotidyl Transferase dUTP Nick End Labeling (TUNEL) Staining

Paraffin-embedded renal tissue sections were prepared and subjected to deparaffinization, washing, hydration and permeabilization. Subsequently, the sections were incubated in TUNEL reaction buffer at 37 °C for 1 h under dark conditions. After washing, counterstaining and mounting were performed. Section images were captured using a digital slide scanner, LG-FS80 (Servicebio, Wuhan, China) to observe and record the apoptosis of renal tissue cells [23].

### 2.12. Proteomic Analysis

For the proteomic profiling, three bio-replicated samples were randomly selected from both the control and F-53B high-dose exposure groups. The total protein was extracted from these samples. The aliquots of the extract were used to quantify protein and detect SDS-PAGE, and the remainder was digested by trypsin. After desalting the digested peptide segments, the samples were analyzed by liquid chromatography-tandem mass spectrometry (LC-MS/MS) using the Data-Independent Acquisition (DIA) method [24]. The acquired data were processed for matching to spectral libraries, quantitative extraction and subsequent statistical analysis.

### 2.13. Statistical Analysis

Normality of data distribution was assessed using the Shapiro–Wilk test, and homogeneity of variances was verified via Levene’s test. Normally distributed continuous variables were expressed as mean ± standard deviation (SD), whereas non-normal continuous variables were presented as median with interquartile range (IQR). Comparisons of continuous variables between two groups were performed using the independent samples *t*-test for normally distributed data and the Mann–Whitney U test for non-normal data. One-way analysis of variance (One-way ANOVA) and repeated-measures analysis of variance (repeated-measures ANOVA) were adopted for comparisons among three or more groups based on single measurements and repeated measures, respectively. All statistical analyses were performed using IBM SPSS software (Version 21), where the two-tailed *p* < 0.05 was considered statistically significant.

## 3. Results

### 3.1. Effects of F-53B on Water Intake, Food Consumption, Body Weight, and Kidney Weight in Mice

**Figure 1:**
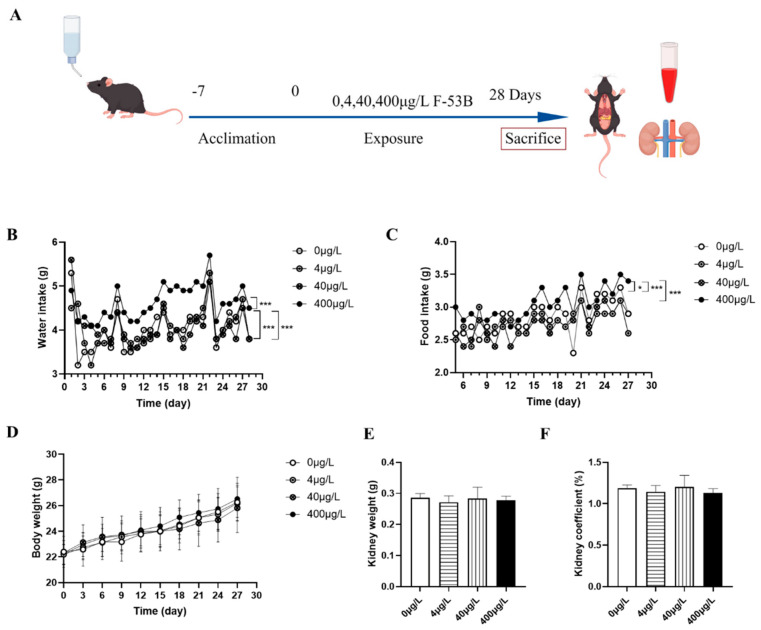
Effects of F-53B exposure on water intake, food consumption, body weight, and kidney weight in mice. (**A**), Experimental treatment design. (**B**,**C**), Changes in water intake and food consumption during the exposure. (**D**) Body weight variation during the exposure. (**E**,**F**), Kidney weight and renal index after the exposure. Renal index = kidney weight (g)/body weight (g) × 100%. All data were analyzed using One-way ANOVA (**E**,**F**) and repeated-measures ANOVA (**B**–**D**) for comparisons among three or more groups, *n* = 10–11 per group (in (**B**), significant differences were observed between 0, 4, and 40 μg/L versus 400 μg/L, *** *p* < 0.001. In Figure (**C**), 0 μg/L compared with 400 μg/L showed * *p* < 0.05; 4 μg/L and 40 μg/L compared with 400 μg/L showed *** *p* < 0.001).

Compared with the control group and the low- and medium-dose groups, mice in the high-dose F-53B group exhibited increased water and food intake (Figure 1B,C). There was no statistically significant difference in final body weight among the groups (Figure 1D). At the end of the experiment, absolute renal weight and renal organ coefficient (renal weight/body weight ratio) were determined. No statistically significant differences in these two indices were detected across all experimental groups (Figure 1E,F).

### 3.2. Effects of F-53B Exposure on Renal Histopathology

**Figure 2:**
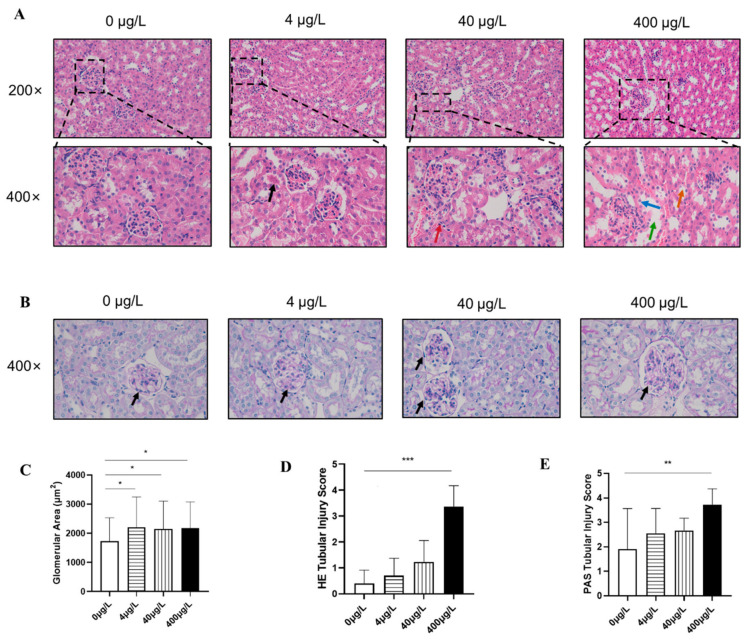
Effects of F-53B exposure on renal histopathology and kidney function in mice. (**A**) H&E staining results. Representative micrographs illustrate alterations, which included infiltration of interstitial inflammatory cells (predominantly lymphocytes, red arrows), formation of intratubular casts (black arrows), dilation of focal tubular (green arrows), removal of proximal tubular epithelial cells (blue arrows) and loss of brush border (orange arrows). (**B**) PAS staining results (400×). (**C**) Quantification of glomerular area. (**D**) Tubular injury scores based on H&E staining. (**E**) Pathological scores based on PAS staining. One-way ANOVA was used for comparisons among the four groups (*n* = 10–11 per group; *, **, and *** indicate statistically significant differences between the treatment group and the control group. * *p* < 0.05, ** *p* < 0.01, *** *p* < 0.001).

Histopathological examination of renal tissues revealed varying degrees of pathological damage in the kidneys of mice across all *F-53B exposure* groups. Representative micrographs (Figure 2A) illustrate these alterations, which included infiltration of interstitial inflammatory cells (predominantly lymphocytes, red arrows), formation of intratubular casts (black arrows), dilation of focal tubular (green arrows), removal of proximal tubular epithelial cells (blue arrows) and loss of brush border (orange arrows). PAS staining further confirmed glomerular damage, characterized by thickening of the glomerular basement membrane (black arrows) and the expansion of the mesangial matrix deposition (Figure 2B). Semi-quantitative analysis showed that exposure to environmentally relevant doses of F-53B resulted in a significantly enlarged glomerulus area and a significantly increased H&E and PAS score for renal impairment (Figure 2C–E).

### 3.3. F-53B Exposure Alters Renal Function and Kidney Injury Markers in Mice

**Figure 3:**
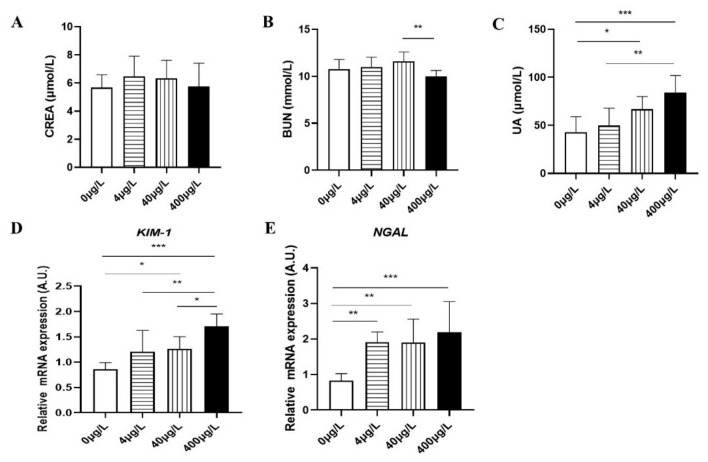
The impact of exposing F-53B on mouse renal function and kidney injury markers (**A**) CREA levels. (**B**) BUN levels. (**C**) UA levels. (**D**) mRNA expression levels of KIM-1 in kidney tissue. (**E**) mRNA expression levels of NGAL in kidney tissue. One-way ANOVA was used for comparisons among the four groups (*n* = 8 per group; all *, **, and *** indicate statistically significant differences between the treatment group and the control group, * *p* < 0.05, ** *p* < 0.01, *** *p* < 0.001). All PCR experiments in this study were performed with 10–11 biological replicates and one technical replicate; all WB assays were conducted using six biological replicates and two technical replicates.

UA, among the typical renal function biomarkers, showed a significant dose-dependent effect following F-53B exposure (Figure 3C). In contrast, creatinine (CREA) and blood urea nitrogen (BUN) levels did not show a significant change, although a decreasing trend was observed in the high-dose group (Figure 3A,B). To further evaluate renal injury, we analyzed the mRNA expression of two sensitive and clinically relevant markers of early kidney injury: The Kidney Injury Molecule-1 (KIM-1) and the Neutrophil-Gelatinase-Associated Lipocalin (NGAL). The quantitative PCR results manifested significant increases in both KIM-1 and NGAL transcript levels in all exposure groups (Figure 3D,E), which confirm the presence of molecular-level subclinical renal impairment.

### 3.4. Proteomic Profiling Reveals Key Pathways in F-53B-Induced Nephrotoxicity

**Figure 4:**
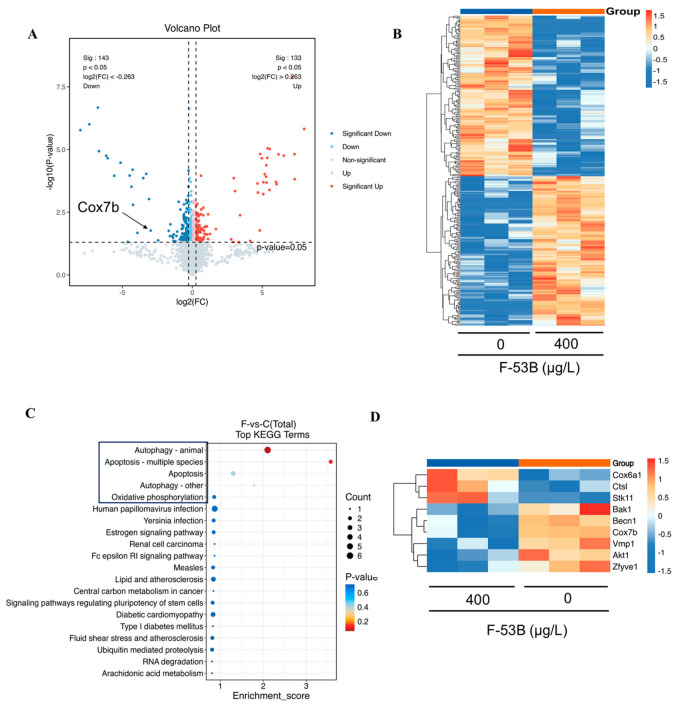
The impact of F-53B exposure on the proteomics of mouse kidney tissue: (**A**) Volcano plot. (**B**) Heatmap of DEPs in mouse kidney tissue after F-53B exposure. (**C**), KEGG analysis of DEPs (Top pathways are autophagy, apoptosis, and oxidative phosphorylation). (**D**) Heatmap of proteins related to oxidative phosphorylation, autophagy and apoptosis [*n* = 3 per group; aforementioned results are based on comparison of the high-dose group (400 μg/L) and the control group (0 μg/L)].

To elucidate the mechanisms underlying renal injury induced by F-53B, a comparative proteomic analysis of renal tissues was performed between the control and high-dose groups (*n* = 3 per group). Using |Fold Change| > 1.2 and *p* < 0.05, 276 differentially expressed proteins were identified, consisting of 133 upregulated and 143 downregulated proteins in the exposed group (Figure 4A,B). In-depth proteomic analysis revealed that Cytochrome c Oxidase Subunit 7b (Cox7b) was the core differentially expressed protein among all downregulated proteins. Its location in the volcano plot is shown in Figure 4A. Cox7b was predominantly enriched in the oxidative phosphorylation pathway related to metabolism; therefore, we focused on this pathway. In addition, Kyoto Encyclopedia of Genes and Genomes (KEGG) analysis indicated that the differentially expressed genes were also enriched in the apoptosis and autophagy signaling pathways. Furthermore, we found that proteins associated with these three pathways, including Cytochrome c Oxidase Subunit 6a1 (Cox6a1), Bcl-2 homologous antagonist/killer (Bak), procathepsin L (Ctsl), serine/threonine-protein kinase (STK11), and autophagy-related protein Beclin1 (Becn1), were significantly altered following F-53B exposure (Figure 4D). Notably, owing to the limited sample size and omics sequencing performed only on samples from two experimental groups, the mechanistic conclusions derived from omics analyses in the present study are exclusively applicable to the high-dose exposure condition.

### 3.5. Proteomics Core Differential Protein Screening and Validation

**Figure 5:**
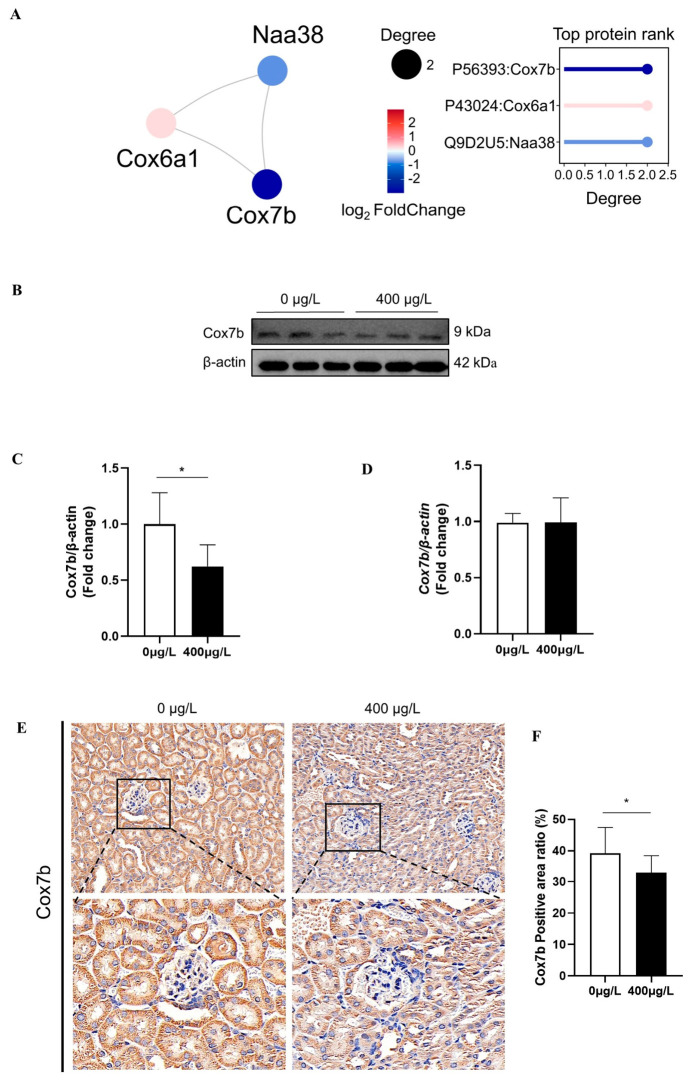
Proteomics core differential protein screening and validation. (**A**) Protein–Protein Interaction Network (PPI) of Cox7b-related proteins; (**B**–**D**) Protein and mRNA expression and quantitative analysis of Cox7b in renal tissues of mice; (**E**,**F**) Immunohistochemical assessment of Cox7b protein expression and quantitative analysis. The independent-samples *t*-test was used to compare the two groups (*n* = 3 per group). * *p* < 0.05, which indicates statistically significant differences between the high-dose group (400 μg/L) and the control group (0 μg/L). All PCR experiments in this study were performed with 10–11 biological replicates and one technical replicate; all WB assays were conducted using six biological replicates and two technical replicates. The original WB images can be found in the Supplementary Materials.

Core differential protein Cox7b was screened out through comprehensive consideration of fold-change, *p*-value, and expression level. (Figure 5A). Expression of Cox7b protein was examined by Western blotting to confirm the proteomic findings. Results showed that Cox7b levels were significantly decreased after exposure to F-53B, but no significant changes in mRNA expression were observed (Figure 5B–D). Microscopic observation revealed that Cox7b is predominantly localized in renal tubular epithelial cells and that expression of Cox7b tends to decrease after exposure to F-53B (Figure 5E,F). This decrease in Cox7b, a key component of the mitochondrial respiratory chain, suggests that mitochondrial function in the kidney is impaired after exposure to F-53B.

### 3.6. Changes in Renal Oxidative Stress-Related Indicators Induced by F-53B Exposure in Mice

**Figure 6:**
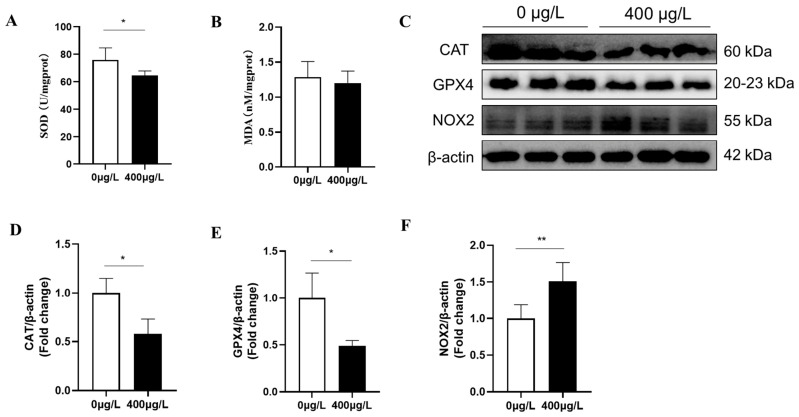
The effect of F-53B exposure on oxidative stress in mice. (**A**) Detection of SOD activity in renal tissue. (**B**) Detection of MDA content in renal tissue. (**C**–**F**) WB detection of the expression of CAT, GPX4, and NOX2 proteins in renal tissue. The independent-samples *t*-test was used to compare the two groups (*n* = 6–8 per group). * and ** indicate statistically significant differences between the high-dose group and the control group; specifically, * *p* < 0.05, ** *p* < 0.01. All PCR experiments in this study were performed with 10–11 biological replicates and one technical replicate; all WB assays were conducted using six biological replicates and two technical replicates. The original WB images can be found in the Supplementary Materials.

The activity of superoxide dismutase (SOD) in the kidneys of F-53B-exposed mice was significantly decreased compared with the control group (Figure 6A,B). In contrast, the malondialdehyde (MDA) concentration showed no significant differences among groups. The expression levels of key antioxidant proteins were also markedly reduced after exposure. Accordingly, the oxidant NOX2 in the high-concentration F-53B group was significantly higher than that in the control group (Figure 6C–F). Collectively, these results indicate that F-53B exposure induces redox imbalance in the kidney tissues of mice.

### 3.7. F-53B Exposure Induces Mitochondrial Perturbation in Mouse Kidney

**Figure 7:**
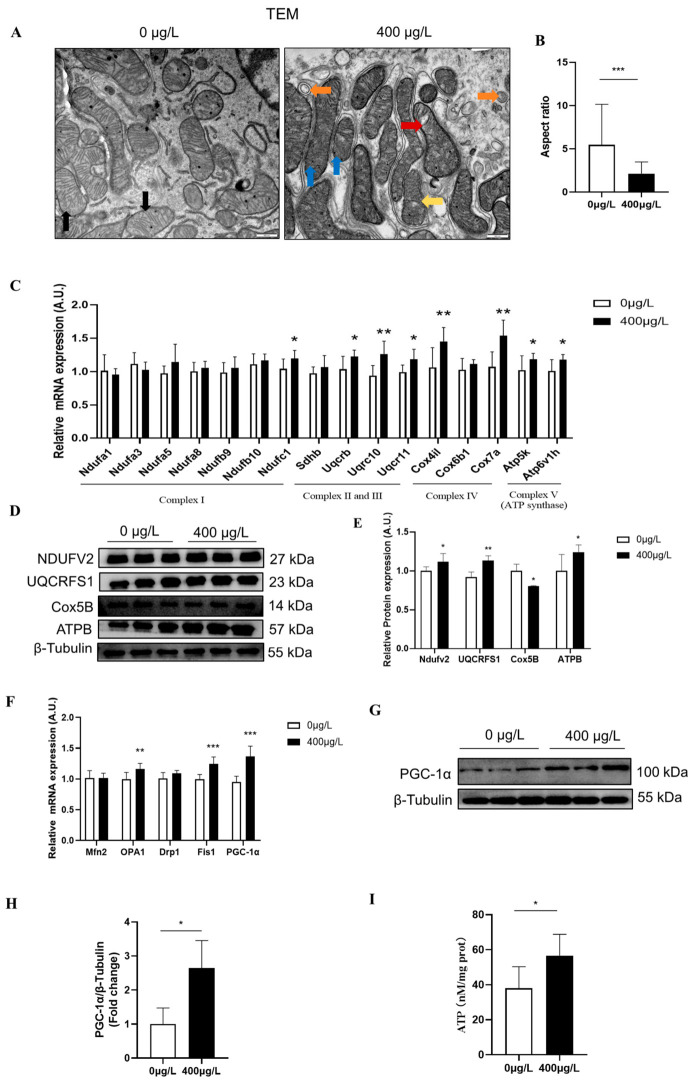
F-53B exposure induces mitochondrial dysfunction in mice. (**A**) Mitochondrial structure under TEM (30,000×, black arrows indicate normal mitochondrial structure, red arrows indicate matrix swelling, yellow arrows indicate mitochondrial fusion, orange arrows indicate donut-shaped mitochondria, and blue arrows indicate blurred or vanished mitochondrial cristae. (**B**) Quantitative analysis of the mitochondrial aspect ratio. (**C**) Expression levels of mitochondrial complexes I, II, III, IV and ATP synthase subunits. (**D**,**E**) Expression and quantitative analysis of representative proteins of different complex subunits and ATP synthase. (**F**) Expression of mitochondrial dynamics genes and biosynthesis genes in mouse renal tissue after exposure. (**G**,**H**) Protein expression and quantitative analysis of mitochondrial biosynthesis gene PGC-1α. (**I**) The effect of F-53B on ATP content in mice. The independent-samples *t*-test was used to compare the two groups (*n* = 6–8 per group). *, ** and *** indicate statistically significant differences between the high-dose group and the control group: * *p* < 0.05, ** *p* < 0.01, and *** *p* < 0.001. All PCR experiments in this study were performed with 10–11 biological replicates and one technical replicate; all WB assays were conducted using six biological replicates and two technical replicates. The original WB images can be found in the Supplementary Materials.

Persistent oxidative stress may induce mitochondrial damage. Transmission electron microscopy (TEM) revealed marked alterations in renal mitochondria following F-53B exposure, characterized by typical pathological changes including mitochondrial swelling, fusion, and disordered cristae structure (Figure 7A). In contrast, mitochondria in the control group exhibited normal morphology with intact and clear cristae. Notably, several mitochondria in the F-53B-exposed group displayed a donut-shaped appearance. Quantitative analysis of mitochondrial aspect ratio showed a significant reduction, which generally indicates mitochondrial fission, swelling, and fragmentation, reflecting structural damage and functional dysfunction of mitochondria.

To further explore the molecular mechanisms underlying mitochondrial dysfunction, we analyzed genes encoding subunits of each component of the mitochondrial respiratory chain and mitochondrial dynamics-related genes, in addition to differentially expressed genes obtained from omics data. The qRT-PCR results demonstrated abnormal upregulation of multiple mitochondrial respiratory chain-related genes, including ubiquinone oxidoreductase subunit C1 (Ndufc1) of Complex I, ubiquinol-cytochrome C reductase binding protein (Uqcrb), ubiquinol-cytochrome c reductase subunit 10 (Uqcr10), ubiquinol-cytochrome c reductase subunit 11 (Uqcr11) of Complex III, cytochrome c oxidase subunit 4 isoform 1 (Cox4i1) and cytochrome c oxidase subunit 7a (Cox7a) of Complex IV, as well as mitochondrial ATP synthase subunit k (Atp5k) of Complex V (Figure 7B).

Furthermore, the expression levels of mitochondrial dynamics-related genes, including optic atrophy 1 (OPA1) and fission 1 (Fis1), together with the mitochondrial biogenesis gene peroxisome proliferator-activated receptor gamma coactivator 1-alpha (PGC-1α), were also significantly increased (Figure 7E). Western blot (WB) analysis confirmed the corresponding changes at the protein level, showing significant alterations in representative subunits of mitochondrial complexes (Figure 7C,D) and in the biogenesis regulator PGC-1α (Figure 7F,G). Finally, detection of ATP content in mouse renal tissues revealed abnormally elevated ATP production in F-53B-exposed mice (Figure 7H). Collectively, these structural, molecular, and functional findings indicate that F-53B exposure leads to mitochondrial perturbation in mouse renal tissues. Notably, although statistically significant, the biological effects of F-53B were relatively modest.

### 3.8. F-53B Exposure Induces Dysregulated Autophagy in Mouse Kidneys

**Figure 8:**
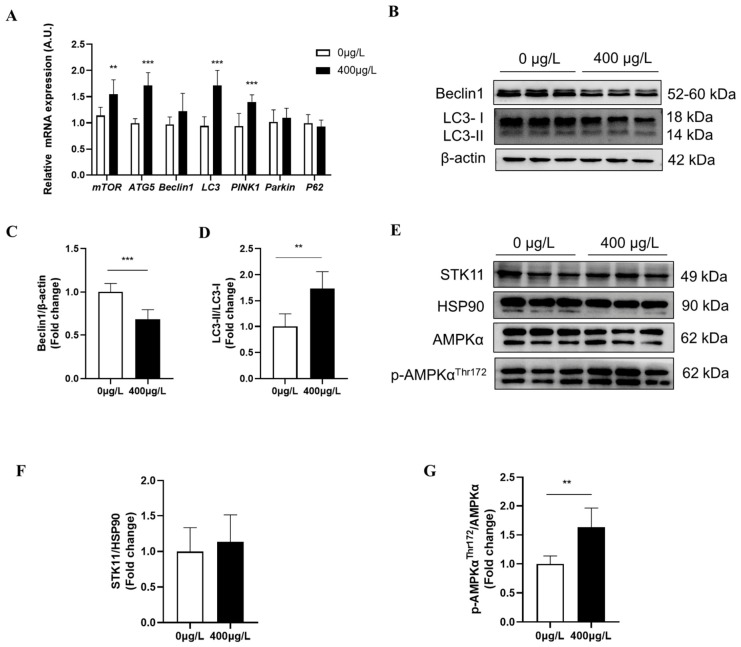
F-53B exposure leads to the activation of autophagy in the renal tissue of mice. (**A**) Expression of autophagy-related genes in mouse kidney tissue. (**B**–**G**) Expression and quantitative analysis of autophagy proteins in mouse kidney tissue. The independent-samples *t*-test was used to compare the two groups (*n* = 6–8 per group). ** and *** indicate statistically significant differences between the high-dose group and the control group (*n* = 6–8). Specifically, ** *p* < 0.01, *** *p* < 0.001. All PCR experiments in this study were performed with 10–11 biological replicates and one technical replicate; all WB assays were conducted using six biological replicates and two technical replicates. The original WB images can be found in the Supplementary Materials.

To evaluate the autophagy response following F-53B exposure, we examined the expression of key genes and proteins involved in autophagy in renal tissue. Quantitative PCR analysis revealed that mice exposed to F-53B showed significant increases in mRNA levels of *mTOR*, *ATG5*, *Pink1*, and LC3 compared to controls (Figure 8A). This transcriptional activation was further confirmed at the protein level by expression of Beclin1, the ratio of LC3-II to LC3-I (a well-known marker for autophagy), STK11, and p-AMPKα^Thr172^/AMPKα 8B-8G. Nevertheless, statistical significance was not observed for all endpoints. Collectively, these results demonstrate that exposure to F-53B induces autophagic activation in the kidney tissue of mice.

### 3.9. Apoptosis Induced in Mouse Kidney Cells upon F-53B Exposure

**Figure 9:**
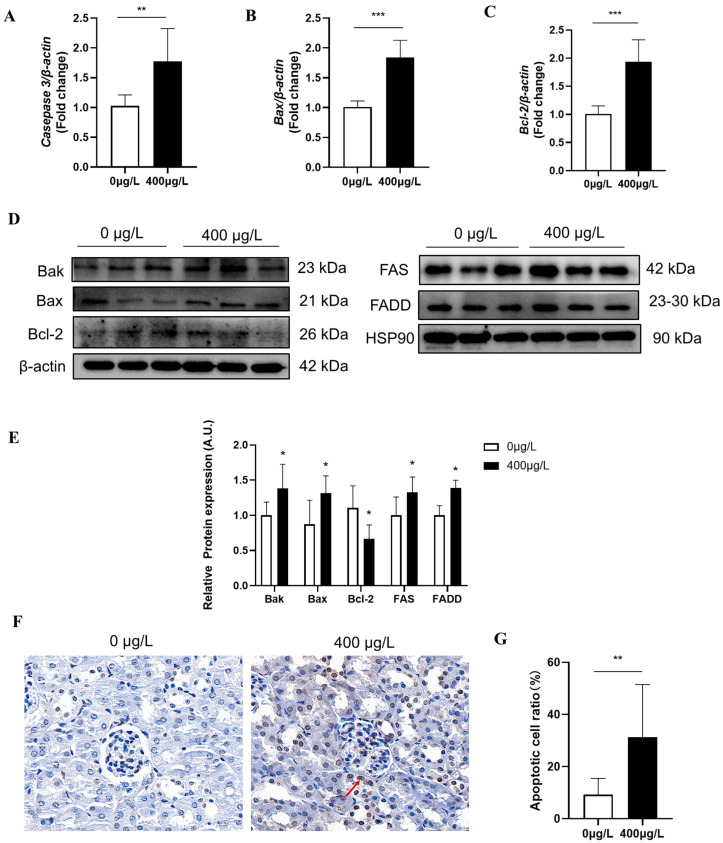
Apoptosis induced in mouse kidney cells upon F-53B exposure. (**A**–**C**) Expression of apoptosis-related genes in mouse renal tissue after F-53B exposure. (**D**,**E**) Protein expression and quantitative analysis in mouse renal tissue. (**F**,**G**) Representative images and relative quantitative analysis of TUNEL staining. Apoptotic cells are marked by red arrows. The independent-samples *t*-test was used to compare the two groups (*n* = 3–8 per group). *, ** and *** indicate statistically significant differences between the high-dose group and the control group; Specifically, * *p* < 0.05, ** *p* < 0.01, *** *p* < 0.001. All PCR experiments in this study were performed with 10–11 biological replicates and one technical replicate; all WB assays were conducted using six biological replicates and two technical replicates. The original WB images can be found in the Supplementary Materials.

To further validate our proteomic findings regarding apoptosis activation, we evaluated apoptotic markers in renal tissues. qRT-PCR analysis demonstrated significantly upregulated expression of the apoptosis-related genes Bax, Caspase3, and Bcl-2 in mice exposed to F-53B (Figure 9A–C). These transcriptional alterations were further confirmed by Western blot (WB) analysis (Figure 9D,E). WB results showed marked increases in the levels of the mitochondrial apoptotic pathway proteins Bak and Bax, whereas the expression of the anti-apoptotic protein Bcl-2 was distinctly decreased. In addition, the expression of the extrinsic apoptotic proteins FAS/FADD was also significantly upregulated. TUNEL staining results revealed that the proportion of apoptotic renal cells was significantly increased in mice of the high-dose group. These pronounced changes in apoptotic regulators indicate that the apoptotic pathway is activated in renal cells following F-53B exposure.

## 4. Discussion

Chlorinated polyfluoroalkyl ether sulfonate F-53B is increasingly being produced and utilized as a replacement for perfluoroalkyl-octane (PFOS), which is leading to persistent accumulation in various environmental compartments. Due to its protein-affinity, F-53B readily interferes with the physiology of the kidneys, making the kidneys (the vital organ for excretion and homeostasis) particularly vulnerable to its adverse effects. Although F-53B has been shown to be hepatotoxic [25,26,27], neurotoxic [28], and toxic to the developing fetus [29,30,31], and a cause of intestinal toxicity [32], its potential nephrotoxicity remains inadequately characterized. Therefore, this study was designed to systematically evaluate the effects on the kidneys of environmentally relevant F-53B concentrations in mice and to elucidate the underlying molecular mechanisms.

The kidneys play a key role in maintaining extracellular fluid volume and osmotic balance by controlling the release of nitrogenous waste, electrolytes and water. Previous studies have reported that potassium chloride supplementation may increase water intake, urine output and urinary excretion of potassium, chloride and nitrogen in adult male mice [33,34]. Consistent with these findings, increased water and food intake were observed in mice exposed to high doses of F-53B (Figure 1B–D). This effect can be attributed, at least in part, to the molecular structure of F-53B, which is a potassium salt. Increased intake of F-53B is likely to increase potassium ion levels and thus stimulate thirst and appetite, a response similar to that seen with potassium chloride supplements. To support this hypothesis, urinary potassium levels showed a significant dose-dependent increase in the exposed groups.

Serum creatinine (CREA) and blood urea nitrogen (BUN) are well-known biomarkers for renal function, and elevated levels are often indicative of renal impairment [35,36]. In a recent community-based study of 278 males, Chen et al. reported that mixtures of per- and polyfluoroalkyl substances (PFAS) were significantly associated with increased uric acid (UA) levels, although no individual PFAS compound was significantly associated with changes in estimated glomerular filtration rate (eGFR). This suggests that exposure to PFAS may not significantly affect glomerular filtration but rather impair the tubular clearance of UA [37]. In support of this, toxicological evidence suggests that exposure to PFAS may cause structural and cellular damage to the renal tubules, thereby impeding clearance of urea nitrogen and promoting hyperuricemia [38,39,40]. Our results align with these observations: uric acid levels in F-53B-exposed mice were increased in a dose-dependent manner, whereas no statistically significant differences were observed in serum creatinine or urea nitrogen levels across exposure groups.

This study further demonstrated that F-53B exposure impairs renal antioxidant capacity in mice. Oxidative stress occurs when the balance between the pro-oxidant and antioxidant systems is impaired by excessive levels of ROS [41]. For instance, previous studies have demonstrated that different doses of F-53B may cause oxidative stress in mice [17,30]. In addition, PFAS can induce biomarkers of oxidative stress such as MDA [42] and superoxide dismutase (SOD, a key enzyme in the endogenous antioxidant defense system) [43]. However, no significant intergroup difference in MDA (a terminal product of lipid peroxidation) levels was observed in the present study, which may be attributed to the following potential explanations [44]. First, MDA is a conventional late-stage oxidative stress biomarker that reflects severe and irreversible lipid peroxidation damage. Mild or early renal oxidative injury may fail to trigger remarkable elevation of circulating MDA; second, the limited exposure time was insufficient to induce persistent and massive lipid peroxidation in renal tissues and peripheral blood, so obvious MDA accumulation was not observed across all groups; third, compensatory endogenous antioxidant systems may neutralize excess ROS in a timely manner, inhibiting the subsequent production and release of MDA. Notably, the significant changes in upstream regulators (SOD, NOX2, CAT, GPX4) suggest that antioxidant defense mechanisms were engaged without resulting in overt lipid damage.

Kidneys, which have a high energy requirement due to their complex filtering and reabsorption functions, possess the highest mitochondrial density and oxygen consumption rate after the heart. Mitochondrial oxidative phosphorylation serves as the primary source of ATP, underscoring the importance of mitochondrial integrity for renal function. Interestingly, our electron microscopy analyses revealed the presence of donut-shaped mitochondria, which is a distinct morphologic marker for mitochondrial dysfunction [45,46]. These abnormal mitochondrial structures have been documented in axonal protrusions of aged monkeys, cultured cells under stress to mitochondria and oocytes and embryos of aged worms [47,48,49,50,51], and are generally considered to be characteristic of mitochondrial dysfunction [45,49].

It is important to note that Cox7b is an important subunit in maintaining the normal structure and function of complex IV in the mitochondrial electron transport chain. Mutation or dysregulation of Cox7b has been associated with tumors and other diseases in skin, blood and nervous system [52,53,54]. In this study, exposure to F-53B resulted in significant Cox7b downregulation, suggesting subsequent damage to the mitochondrial electron transport chain. Our findings also show that exposure to environmentally relevant doses of F-53B results in dysregulated expression of several mitochondrial electron transport chain subunits, including Ndufc1, Uqcrb, Cox7a, and Atp5k, as well as several mitochondrial dynamics and biogenesis factors, with marked upregulation (Figure 7). This pattern may reflect a compensatory reaction which is characterized by transient mitochondrial structural remodeling and slightly elevated energy metabolism, accompanied by increased electron transport and synthesis of ATP, to cope with external stress. This interpretation is consistent with the elevated ATP content observed in the renal tissues of mice exposed to F-53B (Figure 7). However, this compensatory over-regulation may be transitory and inappropriate. Sustained overexpression of respiratory complex subunits may interfere with the stoichiometry of the electron transport chain, increase the electron leakage and ultimately contribute to oxidative damage [55,56]. Thus, initial hyperfunction may be a transient condition that, after prolonged exposure, may progress to energy depletion. A previous study reported that exposure to PFAS mixtures triggers mitochondrial dysfunction in larval zebrafish, characterized by elevated activities of mitochondrial Complex II and Complex III alongside upregulated transcription of genes related to the mitochondrial respiratory chain. Such transcriptional upregulation implies enhanced oxidative phosphorylation to facilitate ATP synthesis [57]. These findings are consistent with the present work; increased expression of relevant subunits presumably reflects a compensatory response aimed at sustaining basal electron transport activity [58]. Nevertheless, the upregulation of genes encoding distinct respiratory complex subunits may exert adverse impacts on mitochondrial respiration, such as accelerated oxygen consumption. Persistent aberrations in ATP homeostasis can ultimately induce loss of cell viability and consequent mitochondrial dysfunction.

Severe mitochondrial damage can trigger the initiation of a specific form of apoptotic mitophagy [59], indicating that mitochondrial damage may activate both autophagy and apoptosis [60]. Following mitochondrial dysfunction, autophagy activation, as indicated by increased LC3-II and p62 degradation, as well as markers of apoptosis, including activation of Caspase-3, modulation of Bak, Bax, and Bcl-2, and involvement of FAS and FADD in the mitochondrial pathway, were also observed (Figure 9). Our results revealed downregulation of Beclin1 and an increased LC3 ratio, which is consistent with suppressed autophagy reported in fibroblasts from patients with Down syndrome [61]. This suggests a potential uncoupling between autophagosome formation and degradation.

In summary, the mechanistic cascade for renal injury induced by F-53B in adult mice is proposed. F-53B initially causes oxidative stress, which in turn causes mitochondrial perturbation. Sustained mitochondrial perturbation then activates both autophagy and apoptotic pathways, ultimately leading to damage to the kidneys. Notably, F-53B-related changes achieved statistical significance yet exhibited only limited biological effect sizes. However, our research has certain limits. Firstly, only control and high-dose groups were included in the proteomic analysis, which hinders clarification of dose-dependent mechanistic differences. Secondly, lacking comprehensive methods, such as mitochondrial membrane potential assay and direct respiratory chain complex activity measurement, results in an incomplete evidence chain. Lastly, this study did not consider gender differences in F-53B nephrotoxicity and focused only on short-term (4-week) exposure, lacking long-term exposure research to assess cumulative renal damage or potential irreversible effects. Therefore, it is essential to employ a broader range of detection techniques in future investigations to strengthen this evidence chain.

## 5. Conclusions

In conclusion, our findings confirm that exposure to F-53B at an environmentally relevant dose poses a risk for renal injury. Proteomic analysis exclusively conducted on the high-dose group put forth a mechanism centered on disrupted mitochondrial oxidative phosphorylation, marked by abnormal elevations in electron transport chain complexes and ATP levels, which triggers autophagy and apoptosis. Statistically significant differences were observed following F-53B exposure, though the overall effect magnitude remained modest. Taken together, this study not only provides novel insights into the molecular mechanisms of F-53B-induced nephrotoxicity but also underscores the potential public health risks associated with this contaminant.

## Figures and Tables

**Table 1:** Sequences of the primers of target genes.

Gene	Forward Primer	Reverse Primer
*KIM-1*	CTGCTGCTACTGCTCCTTGT	GCAACCACGCTTAGAGATGC
*NGAL*	ACGGACTACAACCAGTTCGC	GGGACAGCTCCTTGGTTCTT
*SOD1*	GGGAAGCATGGCGATGAAAG	GGTTCACCGCTTGCCTTCTG
*SOD2*	TGGAGAACCCAAAGGAGAGTTG	GCCTGAACCTTGGACTCCC
*Nrf2*	TGGGCAACCATCACTCTGCT	TCTGCTGCAAGTAGCCTCG
*CAT*	GCCCTGGTCGGTCTTGTAAT	ATGGTCACCGGCACATGAAT
*Ndufa1*	GCGAGTAACGGTGCGGAG	GCAACTCGTTTTTCCTTGCCC
*Naufa3*	GAACAAGATGGCCGGGAGAATC	GACAGAGAAGGACACCACCAG
*Ndufa5*	GGGCTTGCTGAAAAAGACAACT	AACATCTGGCTCCTCGTGTG
*Ndufa8*	TGGGGCAGTTGTCTAAGGTCAC	GTGCTTGGCGGGTTTCAGAT
*Ndufb9*	GGACAAGGTGGAGCGGATAC	GACAGCCATCGGTAGGTACG
*Ndufb10*	GTGACCCTCGTGAGAGAGTT	TGTGATGTCTGGCACTCGAC
*Ndufc1*	TAGTGCTGCGCTCGTTTTCG	TTCGACCGTGTTGAAGAGCAG
*Sdhb*	GACTTCACAGAGGAACGCCT	TCCCCGGATTCAGACCCTTG
*Uqcrb*	TTCAGCATCAAGCAAGTGGC	CATCTCGCATTAACCCCAGT
*Uqcr10*	GGTGACTGGGAGGGAGAAACT	CCCCAACTCCAGGCAAACAG
*Uqcr11*	GGGAACTGGCCAGAAACTGGATT	CCGTTGATGTAAGGCACCCAG
*Cox4i1*	CCTTGGACGGCGGAATG	CGAAGGCACACCGAAGTAGA
*Cox6b1*	CGGGACAATCTTTAGGAGTCAGG	CTGTCAAAGGGGGCAGTTTTG
*Cox7a*	CAGGATCCGGAGTCTTAGAACAG	AGGTCATTGTCGGCCTGGAAG
*Atp5k*	GTCTCTCCACTCATCAAGTTCG	CGCTGCTATTCTCCTCTCCTC
*Atp6v1h*	GGCAGCCAGTGTGCTAAAAC	CACGCTGGTGATTTTCCTGC
*Mfn2*	ACTTCTCCTCTGTTCCAGTTGT	CAGGGACATCTCGCCAGTTT
*OPA1*	TTCTGAGGCCCTTCTCTTGT	TTCTTTGTCTGACACCTTCCTGT
*Drp1*	TAGTGGGCAGGGACCTTCTT	CCATTCTTCTGCTTCAACTCCATT
*Fis1*	CTCCGGTTGATAGACCGCTA	AATTTCCTTTCAAAATTCCTTGCAG
*Casepase3*	GCTTGGAACGGTACGCTAAG	TCCACTGACTTGCTCCCATG
*Bax*	AGACAGGGGCCTTTTTGCTAC	AATTCGCCGGAGACACTCG
*Bcl-2*	GTCGCTACCGTCGTGACTTC	CAGACATGCACCTACCCAGC
*mTOR*	ACCGGCACACATTTGAAGAAG	CTCGTTGAGGATCAGCAAGG
*ATG5*	TGTGCTTCGAGATGTGTGGTT	ACCAACGTCAAATAGCTGACTC
*Beclin1*	GAAACTGGACACGAGCTTCAAGA	ACCATCCTGGCGAGTTTCAATA
*β-actin*	TTCGTTGCCGGTCCACACCC	GCTTTGCACATGCCGCAGCC

**Table 2:** Target antibody information.

Antibody	Company	Article Number
Cox7b	Proteintech, Wuhan, China	11417-2-AP
NDUFV2	ABclonal, Wuhan, China	A7442
UQCRFS1	ABclonal, Wuhan, China	A9517
Cox5B	ABclonal, Wuhan, China	A23762
ATPB	ABclonal, Wuhan, China	A11214
PGC-1α	Proteintech, Wuhan, China	66369-1-Ig
Bax	Proteintech, Wuhan, China	50599-2-Ig
Beclin 1	Proteintech, Wuhan, China	11306-1-AP
LC3	Proteintech, Wuhan, China	81004-1-RR
β-actin	Abcam, Waltham, MA, USA	AB8227
β-Tubulin	Proteintech, Wuhan, China	10094-1-AP

## Data Availability

The original contributions presented in this study are included in the article/Supplementary Materials. Further inquiries can be directed to the corresponding authors.

## Supplementary Materials

The following supporting information can be downloaded at: https://www.mdpi.com/article/10.3390/biom16070938/s1, The original Western Blot images can be found in this section.
