# Supplementary material for: Association of F-53B Nephrotoxicity with Oxidative Stress-Mediated Mitochondrial Dysfunction and Altered Autophagy–Apoptosis Crosstalk
Source: Biomolecules. 2026 Jun 24;16(7):938. doi: 10.3390/biom16070938 (PMC13406552; doi:10.3390/biom16070938)
Supplement: Supplementary file 1 [file biomolecules-16-00938-s001.zip › biomolecules-4367037-supplementary.pdf]

We acknowledge that the submitted original Western blot images do not show visible molecular weight marker bands. This is due to a technical limitation of the chemiluminescence imaging system available in our institution. We guarantee the authenticity and reliability of all presented blots, and the corresponding molecular weight markers will be manually annotated on the images. The prestained protein marker used in this experiment is shown in the figure below.

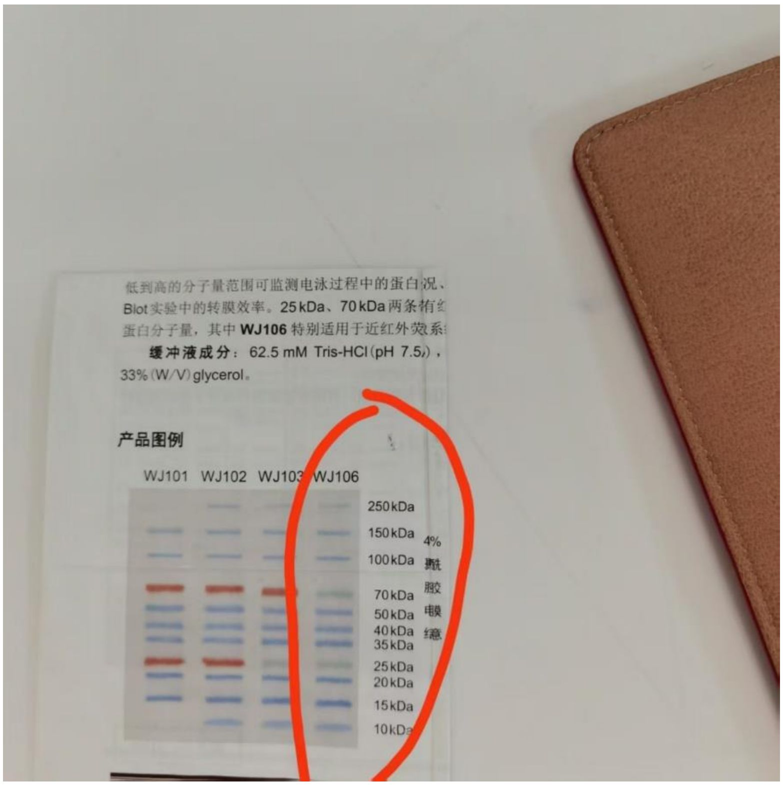

Fig 5B Cox7b β-actin

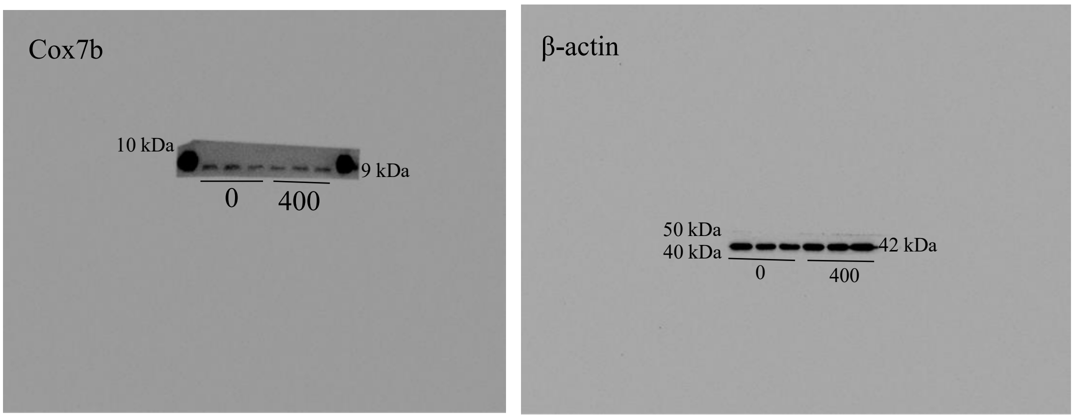

Fig 6C CAT、GPX4、NOX2、β-actin

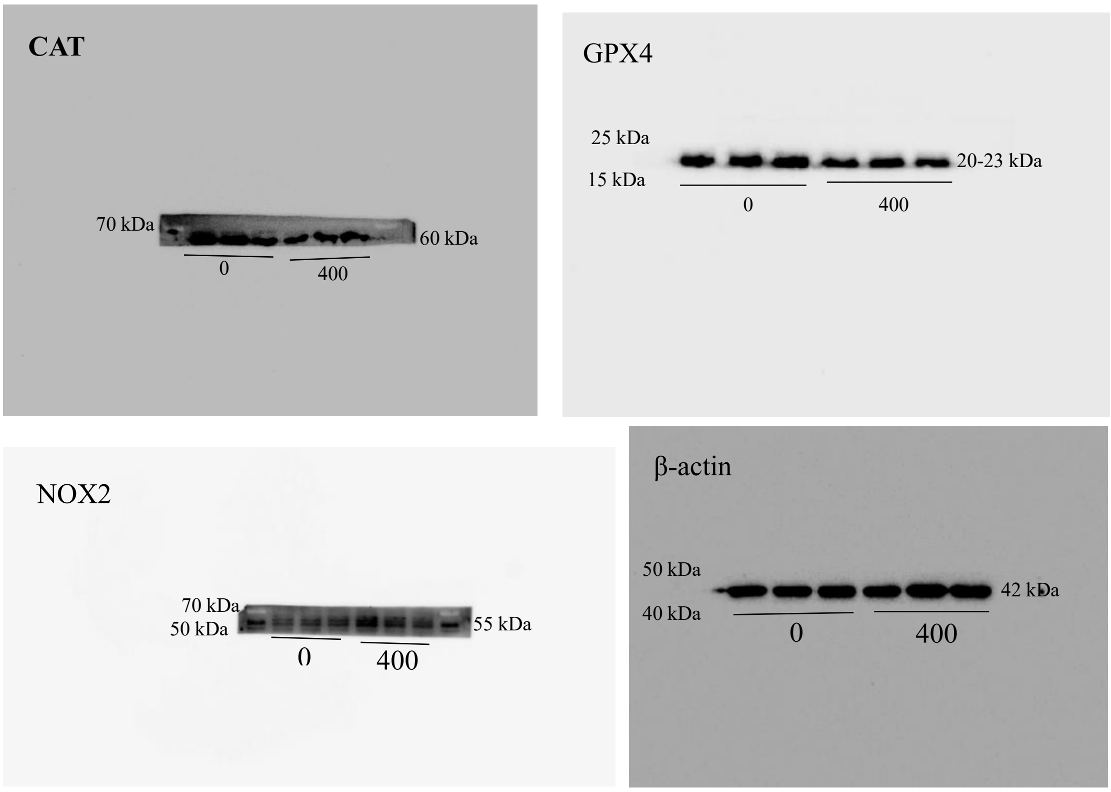

Fig 7D NDUFV2 UQCRFS1 Cox5B ATPB β-Tubulin

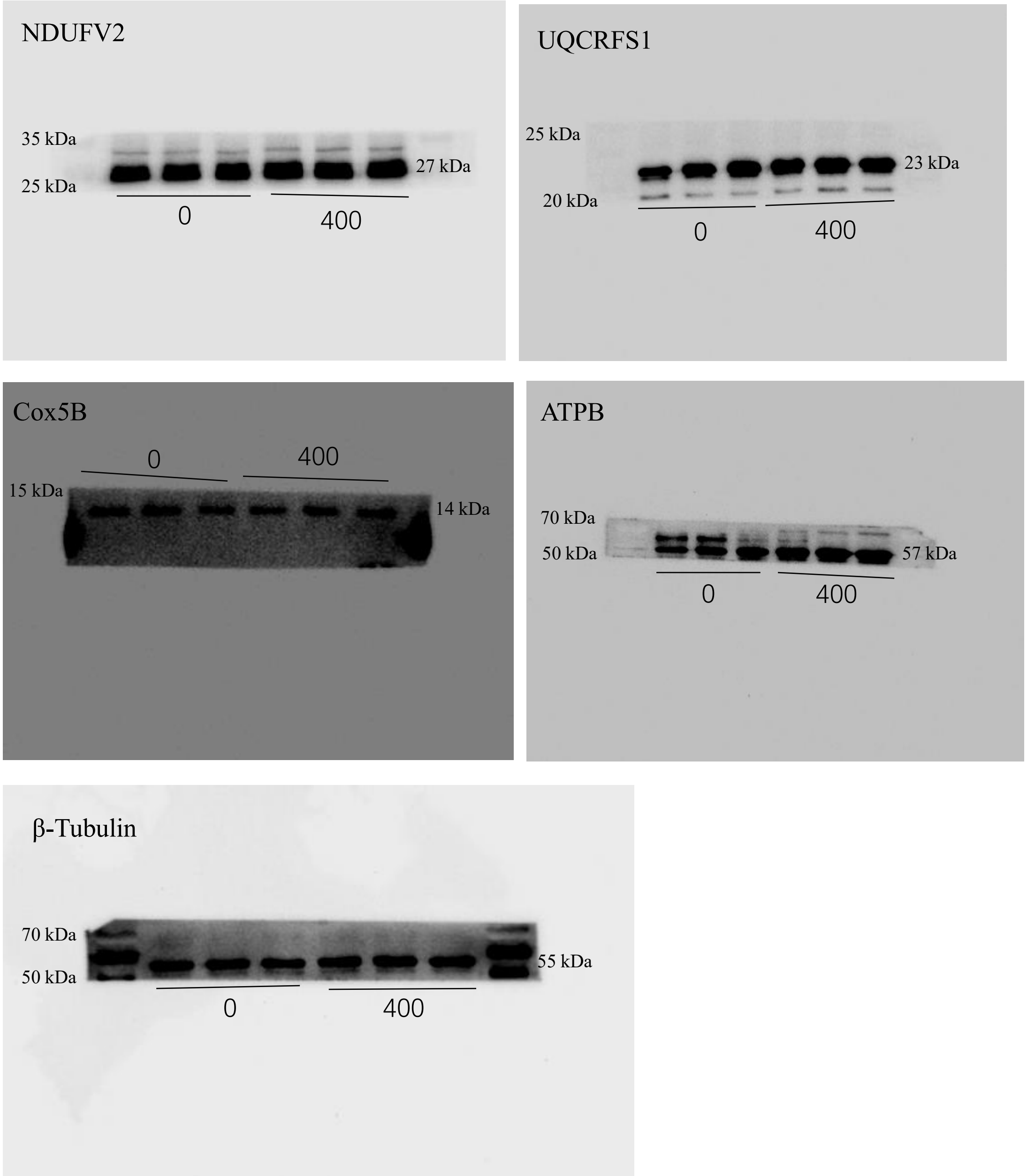

Fig 7G PGC-1α β-Tubulin

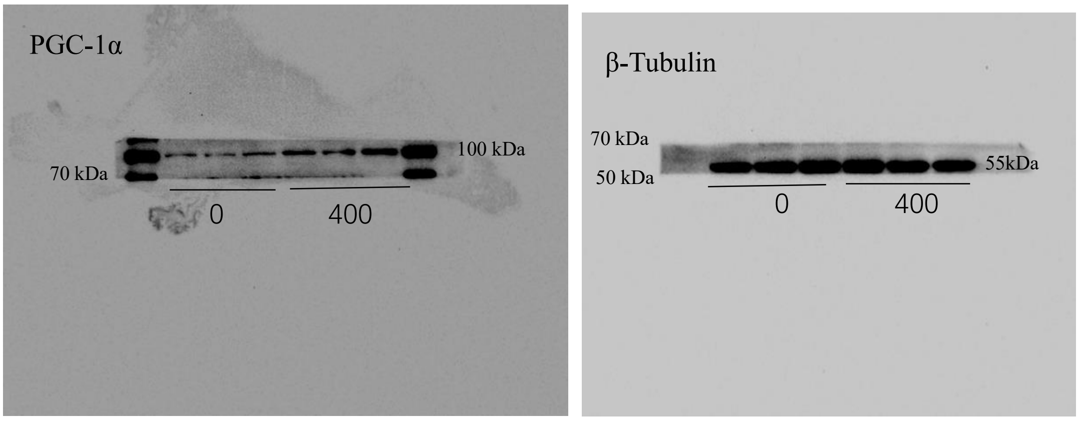

Fig 8B Beclin1 LC3 β-actin

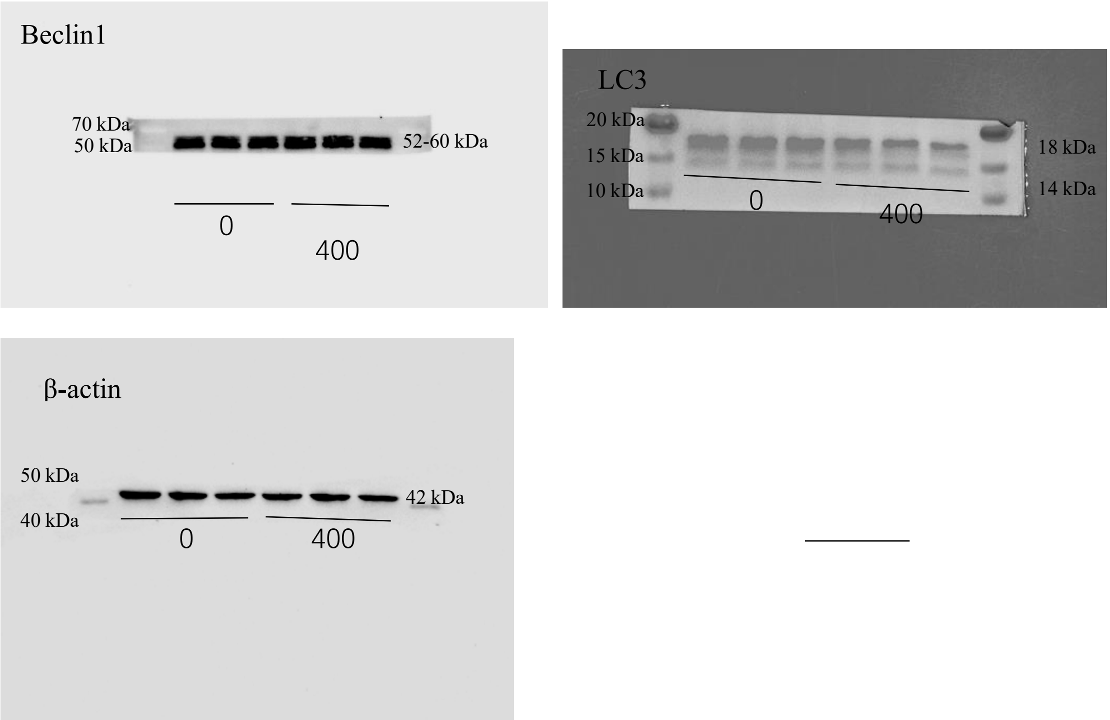

Fig 8E STK11 HSP90 AMPK p-AMPKα

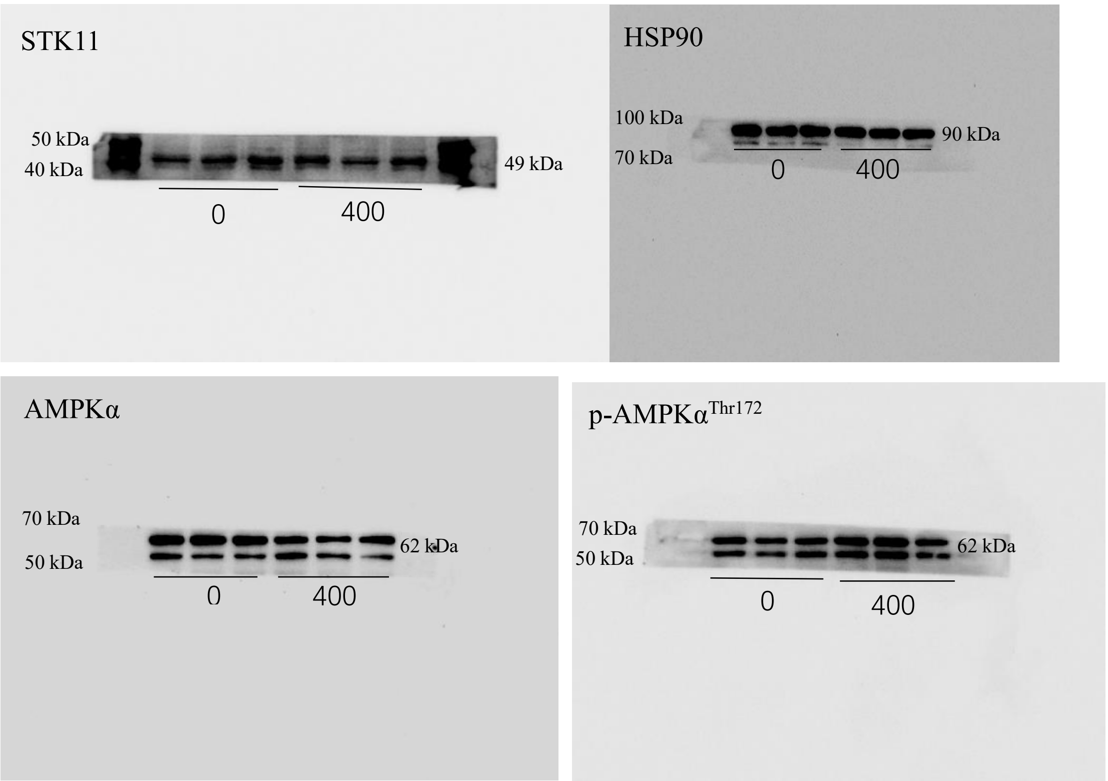

Fig 9B Bak Bax Bcl-2 β-actin FAS FADD HSP90

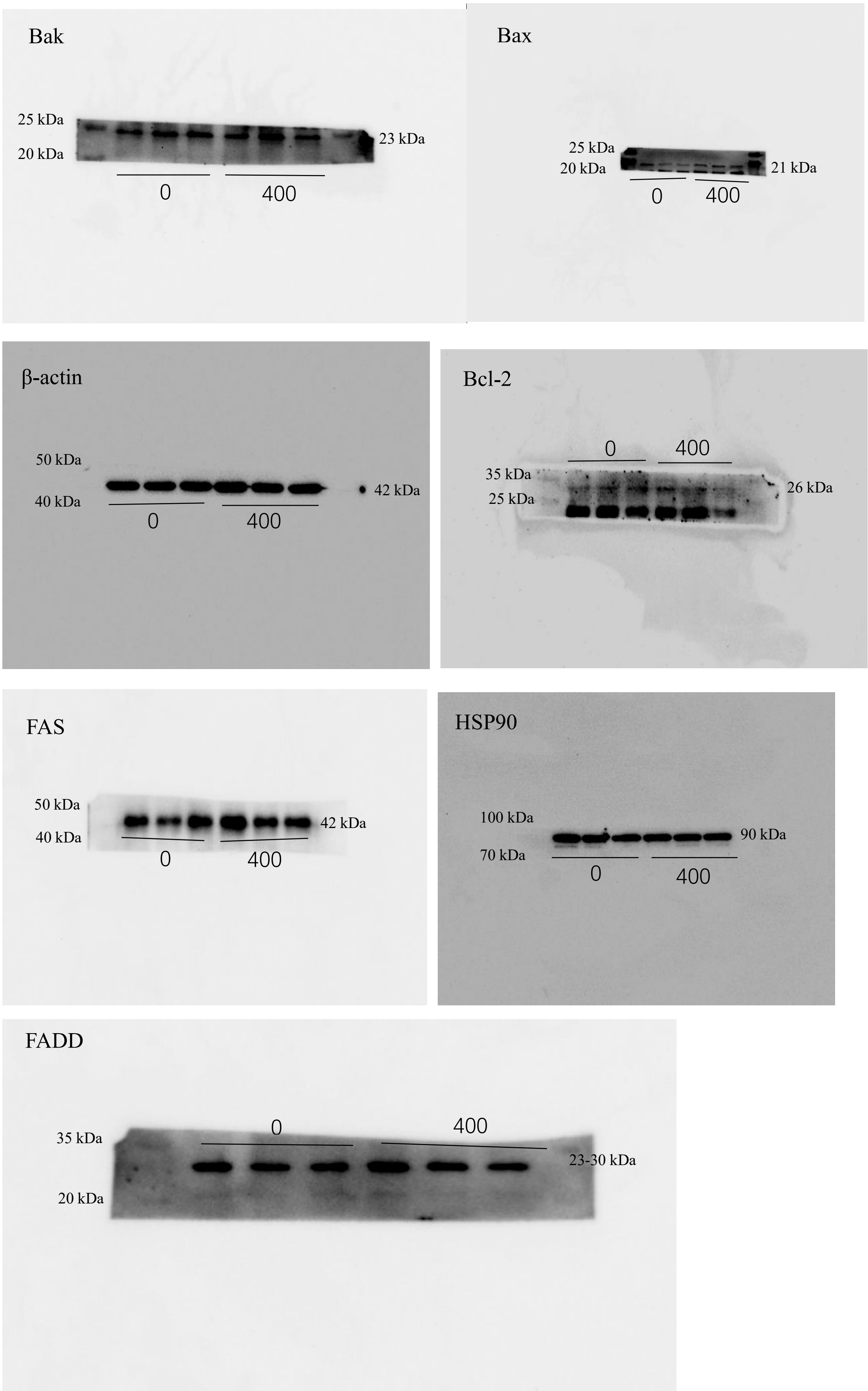

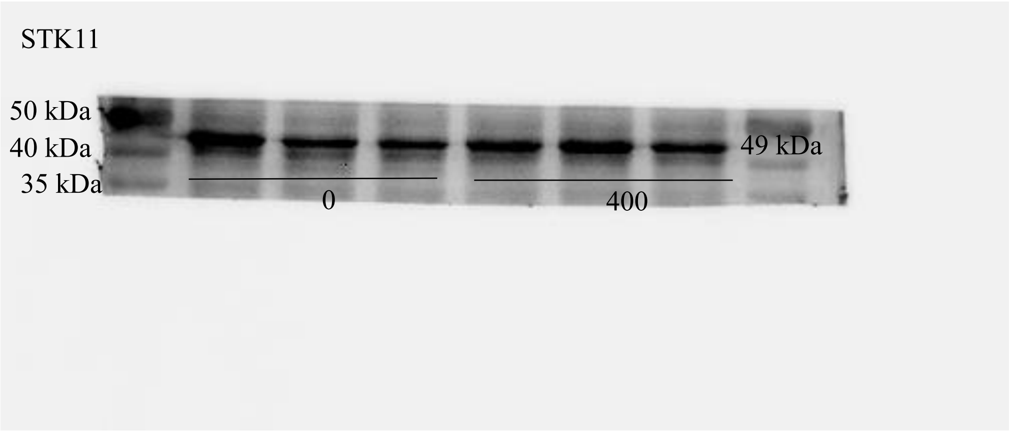
STK11
50 kDa
40 kDa
35 kDa
49 kDa
0
400

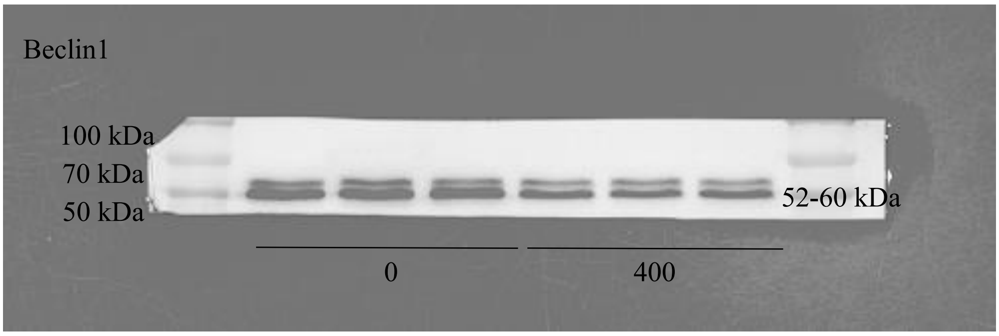
Beclin1
100 kDa
70 kDa
50 kDa
52-60 kDa
0
400
